# Understanding the value of curation: A survey of US data repository curation practices and perceptions

**DOI:** 10.1371/journal.pone.0301171

**Published:** 2024-06-14

**Authors:** Lisa R. Johnston, Renata Curty, Susan M. Braxton, Jake Carlson, Hannah Hadley, Sophia Lafferty-Hess, Hoa Luong, Jonathan L. Petters, Wendy A. Kozlowski

**Affiliations:** 1 Data, Academic Planning & Institutional Research, University of Wisconsin Madison, Madison, Wisconsin, United States of America; 2 Research Data Services, Library, University of California Santa Barbara, Santa Barbara, California, United States of America; 3 University Library, University of Illinois at Urbana-Champaign, Champaign, Illinois, United States of America; 4 University Libraries, University at Buffalo, Buffalo, New York, United States of America; 5 Research and Teaching Services, Library, Princeton University, Princeton, New Jersey, United States of America; 6 University Libraries Center for Data and Visualization Sciences, Duke University, Durham, North Carolina, United States of America; 7 Data Management and Curation Services, Data Services, University Libraries, Virginia Tech, Virginia, United States of America; 8 Research Data and Open Scholarship, Cornell University Library, Cornell University, Ithaca, New York, United States of America; Politecnico di Milano, ITALY

## Abstract

Data curators play an important role in assessing data quality and take actions that may ultimately lead to better, more valuable data products. This study explores the curation practices of data curators working within US-based data repositories. We performed a survey in January 2021 to benchmark the levels of curation performed by repositories and assess the perceived value and impact of curation on the data sharing process. Our analysis included 95 responses from 59 unique data repositories. Respondents primarily were professionals working within repositories and examined curation performed within a repository setting. A majority 72.6% of respondents reported that “data-level” curation was performed by their repository and around half reported their repository took steps to ensure interoperability and reproducibility of their repository’s datasets. Curation actions most frequently reported include *checking for duplicate files*, *reviewing documentation*, *reviewing metadata*, *minting persistent identifiers*, and *checking for corrupt/broken files*. The most “value-add” curation action across generalist, institutional, and disciplinary repository respondents was related to reviewing and enhancing documentation. Respondents reported high perceived impact of curation by their repositories on specific data sharing outcomes including usability, findability, understandability, and accessibility of deposited datasets; respondents associated with disciplinary repositories tended to perceive higher impact on most outcomes. Most survey participants strongly agreed that data curation by the repository adds value to the data sharing process and that it outweighs the effort and cost. We found some differences between institutional and disciplinary repositories, both in the reported frequency of specific curation actions as well as the perceived impact of data curation. Interestingly, we also found variation in the perceptions of those working within the same repository regarding the level and frequency of curation actions performed, which exemplifies the complexity of a repository curation work. Our results suggest data curation may be better understood in terms of specific curation actions and outcomes than broadly defined curation levels and that more research is needed to understand the resource implications of performing these activities. We share these results to provide a more nuanced view of curation, and how curation impacts the broader data lifecycle and data sharing behaviors.

## Introduction

Data curation spans a number of disciplines and can be defined differently according to the various contexts. Yakel [1] observes that data curation falls under the larger umbrella of digital curation, which also encompasses digital preservation, electronic records management, and digital asset management. Likewise, data curation services provided within digital repositories have a lineage to archival science and established standards such as the Reference Model for an Open Archival Information System (OAIS) for the acquisition, processing, ingest, and dissemination of digital objects [2]. Palmer, Weber, Munoz and Renear [3] outline the development of the term “data curation” servicing scholarly communications by differentiating it from similar digitally-based curatorial efforts that are not focused on research data. This distinction is reflected by an excerpt from an earlier definition of data curation: “of interest and usefulness to scholarship, science, and education…,” that describes scholarly considerations in addition to maintenance and discovery [4]. For the purposes of our study, we define data curation as *the various actions taken to ensure that data are fit for purpose and available for discovery and reuse*.

Individual repositories have also used varying levels to inform curation processing decisions and actions [5, 6] or to communicate and assess varying curation service cost models [7]. Other fields of digital information management, such as digital preservation, have community-developed levels that have proved to be useful tools for internal planning, assessment, and communication with stakeholders [8]. Understanding current data curation practices is foundational for assessing the value of these practices to realize the goal of Findable, Accessible, Interoperable, and Reusable (FAIR) data [9].

There has been past work on documenting and defining repository curation actions. CoreTrustSeal (CTS, https://www.coretrustseal.org) created a set of requirements from the Data Seal of Approval and the World Data Systems certifications, for certification of repositories that explicitly includes reuse considerations in its glossary definition of data curation: " The activity of managing and promoting the use of data from their point of creation to ensure that they are fit for contemporary purpose and available for discovery and reuse" [10]. This set of core requirements defines a comprehensive assessment framework for repositories, but does not provide much detail on specific curation actions that may be applied by data curators. The Data Curation Network defined a list of curation actions [11] based on numerous glossary sources, and tested these in a survey of North American academic repositories [12] and in focus groups with researchers across six academic institutions [13]. Similarly, a recent study by Lafia et al. [14] classified and defined a schema of data curation actions to examine the actions most frequently performed by ICPSR curators including “initial review and planning, data transformation, metadata, documentation, quality checks, communication, other, and non-curation work.” A number of repositories have published case studies of their data curation pipelines, workflows, and services [5, 15–19] and in some cases proposed curation models to support the sharing of quality data [19, 20]. While this work lays a firm foundation for understanding potential data curation workflows and practices, in many ways current data curation practices remain mostly opaque to those outside the curation process with significant variation in scope and implementation of data curation activities across repositories.

A useful way to operationalize and interrogate the process of data curation is through levels, tiers, or categories. For instance, Lafferty-Hess et al. [21] performed a grouping exercise placing the DCN defined activities into three levels of service as a mechanism for two academic libraries to internally plan services and communicate with stakeholders. The CTS “levels of curation” are defined in the guidance v0.01 as:

- Content distributed as deposited
- Basic curation–e.g., brief checking, addition of basic metadata or documentation
- Enhanced curation–e.g., conversion to new formats, enhancement of documentation
- Data-level curation–as in C above, but with additional editing of deposited data for accuracy [10].

A content analysis of 100 publicly available CoreTrustSeal self-assessments for repositories certified from 2017–2020 showed that 51% of these repositories reported a CTS curation level D, while 27%, 14%, and 1% reported CTS levels C, B, and A respectively, while 7% did not complete this section [22].

The value of research data curation has been asserted [23, 24] as well as demonstrated through a variety of efforts. Research communities within certain disciplines have long-standing efforts to better curate and provide data for reuse (e.g., social and political science [25], astronomy [26], environmental science [27]). Academic institutions are also increasingly treating research data as a valuable asset that deserves focused management [28]. Particular curation actions involving data citations and persistent identifiers (PIDs) have also been found to enable increased understanding and assessment of the impact of open data publishing [29].

Quantifying the value of curation is a challenge. While theoretical frameworks exist [30, 31] the National Research Council [32] notes: “Precisely how much value curation adds to digital information and for whom are impossible to measure in the absence of an explicit market for digital products with differential pricing for curated and uncurated data.” Recent efforts have been made to better establish the value of research data curation actions. Based on a limited sample of submitted datasets (20 datasets each from four US research university-based repositories), Koshoffer et al. [33] “found the curation process may have had a measurable impact on the metadata captured and did result in more documentation especially the inclusion of readme files, with a dataset submission.” Hemphill et al. [34] analyzed data usage associated with 380 studies in ICPSR, and found that enhancing the record with more keywords was positively correlated with data downloads, as was data published by an institution (as opposed to a principal investigator), and data produced with external funding.

There is burgeoning research that suggests that data curation plays an important role in addressing data quality, leading to better, more valuable and reusable data products. However, there is less clarity on the specificity of data curation activities and practices within repositories and which aspects of our curation workflows add the most value. To address some of these questions representatives of institutions in the Data Curation Network (https://datacurationnetwork.org) developed a survey instrument to capture the level of data curation, specific data curation activities performed by a data repository, and the perceived value-add that curation has on the data sharing process. Our survey inquired about 27 specific data curation actions, informed by published workflows and a scan of the literature, that might be taken to ensure that data are fit for purpose and available for discovery and reuse. Survey questions were informed by several literature sources, a content analysis of repository CoreTrustSeal self-assessments, and the published workflows from data repositories within and outside of the Data Curation Network.

Our survey was promoted to staff and directors at data repositories based in the United States, open for three weeks in January 2021. In this paper we frame our survey results—including 95 individual observations of the levels of curation reported in practice across 59 data repositories in the United States—within the context of past studies and tools that aim to operationalize data curation. We discuss the limitations of the study and propose ideas on how to expand this work to other national contexts. This research will better enable data repositories to benchmark curation actions in a meaningful way and to understand the potential value proposition of data curation as perceived by members of the data curation community.

## Materials and methods

To understand the current practice of US-based data repositories and begin to determine which aspects of our ingest, appraisal and curation workflows add the most value, the authors developed a survey instrument to address the following research questions:

1. What level of curation do repositories provide?
2. What is the perceived value-add that curation has on the data sharing process?

### Instrument development

Adapting from the CoreTrustSeal levels of curation and the FAIR principles [9, 35] our survey instrument defines five levels of curation (Table 1). Our instrument branches for staff/directors of repositories to detail how often 27 curation actions are taken at their repositories [36]. For example, do curators “review metadata” and do they “edit metadata for accuracy.” The curation actions defined in this survey were based on a literature review of the published workflows from well-established repositories, direct experience from the authors who represent 8 different data repositories within the Data Curation Network, and a content analysis of 100 CoreTrustSeal repository self-assessments certified between 2017–2020 [22].

**Table 1:** Levels of curation definitions, examples and abbreviations used for analysis.

Abbreviation	Curation Level	Example actions
L0	Data are distributed as deposited	
L1	Record-level curation	Perform brief metadata checks for increased findability.
L2	File-level curation	Review file arrangement and perform file format conversions for increased accessibility.
L3	Documentation-level curation	Review documentation and request/add missing information for increased reusability.
L4	Data-level curation	Open files and review data contents and may annotate/edit the data for accuracy or interoperability.

Adapted from CoreTrustSeal “Level of Curation Performed” [35] and the FAIR Principles [9].

### Recruitment and distribution

We developed an online survey with 20 questions that use a mix of multiple selection, exclusive selection, Likert-scale, ranking, and open-ended questions. Respondents were first asked to complete the survey for a specific repository, defined by a URL to support name disambiguation.

Distributed via Qualtrics and classified as exempt by the University of Minnesota Institutional Review Board, participation in the survey was voluntary, and consent was informed via a written disclaimer regarding data sharing and confidentiality; participant acknowledgement was implied by beginning the survey. The survey remained open for three weeks (January 4–22, 2021). The target population was repository staff and directors, who were recruited through direct emails to repositories and announcements on data curation and related listservs during the open period. Our goal was to contribute to understanding the community of practice around data curation in repositories. A survey targeting repository end users was conducted separately [37]. Even so, end users were allowed to participate in the survey if our distribution reached them as most survey questions were designed to collect respondents’ perceptions about the value and impact of data curation. We sought inclusiveness of all affiliations and did not to limit participation to one response per repository. Respondents were asked to identify which repository their survey responses considered, and their affiliation with that repository (staff, director, depositor or unaffiliated); as such, individuals may have been identifiable to the authors. Published data was deidentified before sharing.

Even though US data repositories are well represented in registries such as re3data.org or datacite.org, attempts at securing a list of email contacts was unsuccessful. We therefore adopted a non-probabilistic sampling approach via email recruitment on multiple listservs including Research Data Access and Preservation (RDAP), DataCure, DataLibs, International Association for Social Science Information Service and Technology (IASSIST), NIH Data Science, as well as emails to Research Data Alliance (RDA) working groups and DCN repository staff. We acknowledge that our survey distribution method does not allow for a calculable response rate since the total distribution reach is unknown.

### Data analysis

Data analysis for closed-ended questions was carried out on SPSS version 26. We performed descriptive statistics and frequencies along with cross tabulations and Mann-Whitney U-Tests to compare perceptions and opinions about the value of data curation among and across groups.

Free-text responses were examined by two independent coders following an inductive thematic analysis approach [38]. An iterative joint analysis of the initial, independently assigned codes was performed seeking harmonization of codes and their definitions.

## Results

The survey received a total of 120 responses. However, 22 responses were for non-US repositories and three did not provide a repository of reference, therefore this analysis and report is based on a subset of 95 responses for US-based repositories. The majority of the participants self-identified as staff members with 54.7% staff and 35.8% repository directors. The remaining were unaffiliated users (5.3%), and unaffiliated depositors (4.2%).

### Repository demographics

Of the 95 responses, our sample includes 59 unique repositories geographically distributed across 23 different U.S states (S1 Appendix). Among these unique repositories, 11 are members of the Data Curation Network and 10 are certified by CoreTrustSeal. As previously mentioned, our survey did not restrict the number of responses by repository yet the majority of responses (n = 48, 81%) were exclusive; for the remaining 11 repositories we received between 2–17 responses.

Our survey received an equal number of 44 responses each (46.3%), associated with disciplinary data repositories and institutional data repositories. The remaining seven responses (7.4%) were for generalist data repositories. We anticipated the need to evaluate responses this way due to their unique curation needs and challenges. Disciplinary repositories tend to curate a homogenous type of data (e.g., GenBank curates DNA sequences) and curatorial staff at disciplinary repositories may specialize their curatorial workflows to data types unique to that domain. Institutional and generalist repositories, on the other hand, handle heterogeneous types of data from all domains (e.g, from chemical spectra to medieval manuscripts) and therefore must scale their staff time, and potentially their curatorial processes, to accommodate a larger diversity of data challenges.

Survey sampling and distribution was not strategized in such a way to allow us to draw specific comparisons across and between repositories, nor was the purpose of this research to rank unique repositories according to their curation efforts and standards. Instead, most questions were designed to capture respondents’ self-declared perceptions and opinions rather than measuring actual curatorial behaviors and repositories’ performance. Because of this, we treated each answer at a case level and chose not to perform weighting or other statistical techniques which could arbitrarily skew the data or produce misleading results.

### Levels of curation

**Fig 1:**
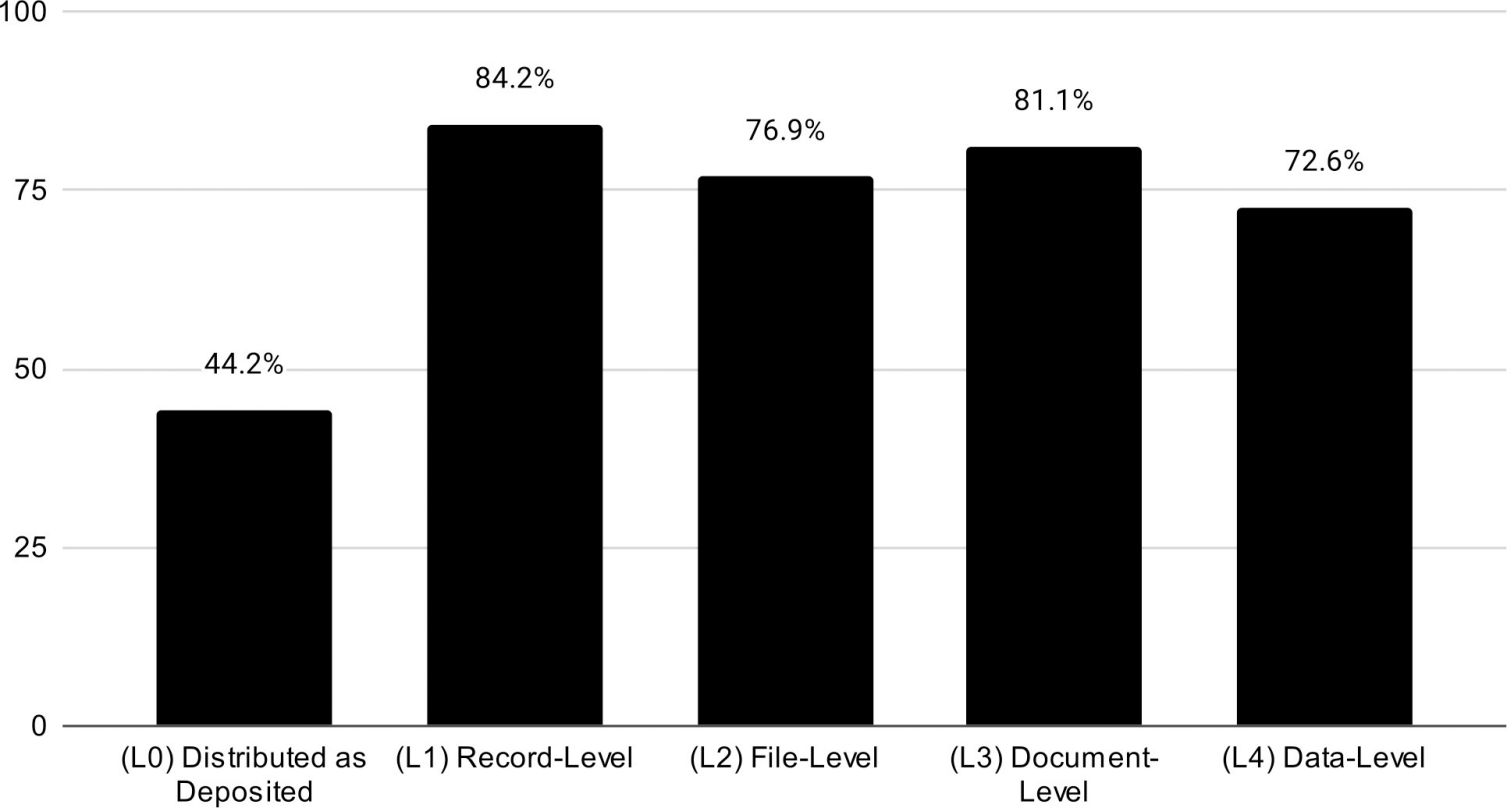
Levels of curation provided at responding repositories, as percentages of total respondents.

For the 94 valid cases for Q3 “Which level(s) of curation does this repository provide?”, which allowed multiple responses, 84.2% of respondents believed their repositories offered Record-level curation (L1), followed closely by Documentation-level (L3, 81.1%), then File-level (L2, 76.9%) and Data-level (L4, 72.6%). Nearly half of respondents also indicated that data are distributed as deposited (L0, 44.2%), as shown in Fig 1.

We also recoded this question as a hierarchy. For example, when a respondent responded they did L1, L2 and L3, we coded this as L3. We found that only a few participants 4.2%, 6.4%, 3.2%, and 12.6% respectively, believe that their repositories of reference offer L0-L3. The great majority (72.6%) of participants reported performing data level curation (L4), indicating that most repositories are reviewing data contents in some capacity.

It is worth noting that we also checked for variance on the data by removing responses from users and depositors, anticipating the chance that they could not be as knowledgeable about all the different levels of curation the repository provides. However, the analysis of the 86 remaining responses from staff members and directors, did not vary much in proportion. For 74.4%, L4 remains the level of curation more often performed, followed by L3 (12.8%), L1 (7%), L2 (3.5) and no curation L0 (2.3%).

All participants were invited to provide additional comments about the level of curation provided by the repository. These results are explored further in the qualitative themes section of this paper. More than half of respondents (55.3%, n = 52) provided a more nuanced glimpse at perceived curation practice for the repositories in our sample. For example:

“Realistically, level of curation varies with discipline, complexity of the dataset, and objective of the depositor. But our overall aim as a repository is to make the data comprehensible and reusable in some meta-analyses.”

    *- Institutional data repository staff member*

“Within certain activity categories, some actions are always performed (e.g., data files are always opened and previewed, saved in various formats) but others are rarely or never performed (e.g., edits to the data is almost never done).”

    *- Disciplinary data repository staff member*

**Fig 2:**
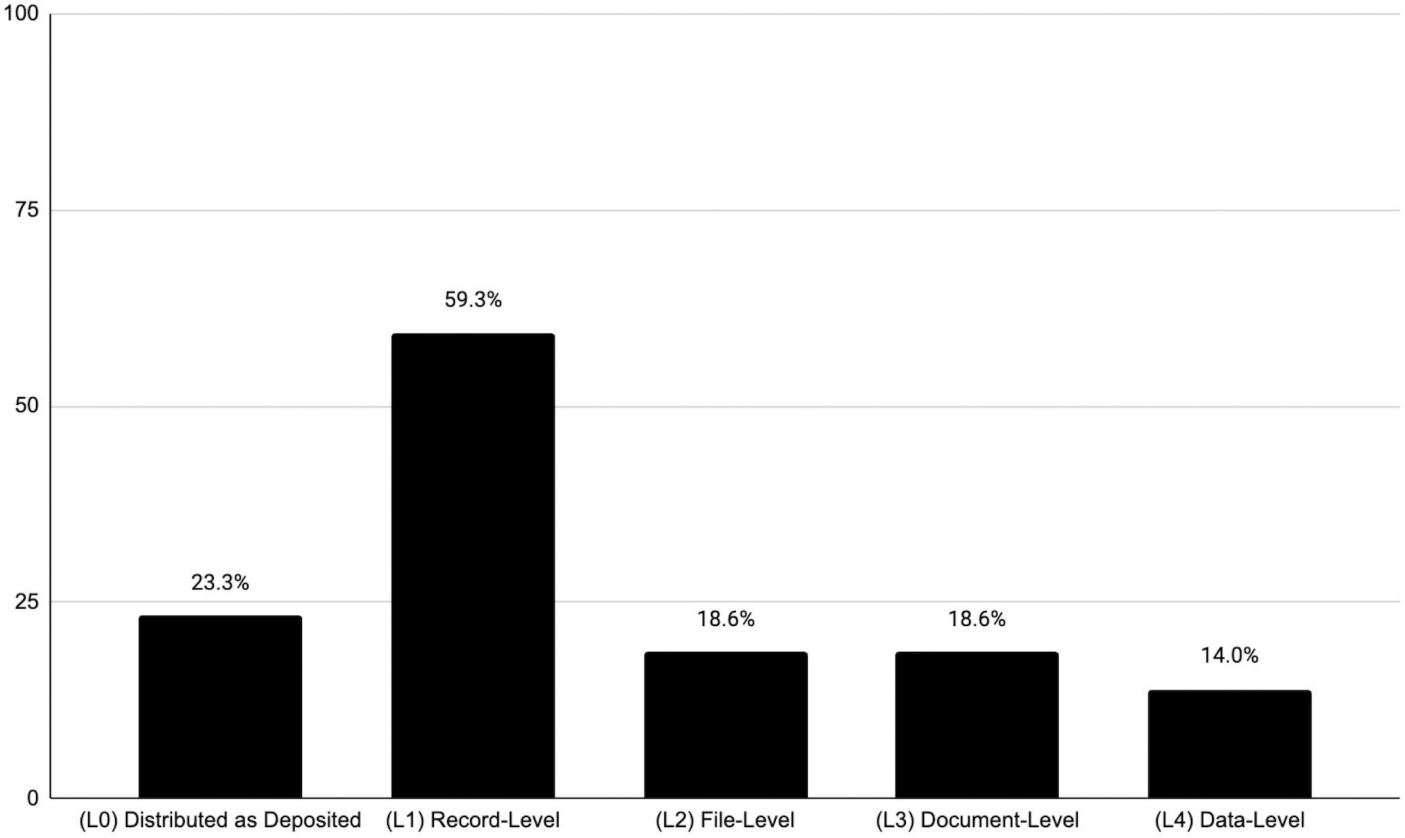
Percentage of staff and directors that reported levels of curation performed at least half of the time, relative to total cases (n = 86).

The subset of staff and directors (n = 86 or 90.5%) were also asked to report the frequency with which they believe each of the levels of curation is applied by their repository of reference, on a scale 0–10. We computed frequencies ranging from the central point “about half of the time” (5) to “most of the time” (10) and calculated their percentage in relation to the total number of valid responses per item and to all 86 cases. As seen in Fig 2, staff and directors perceive that the record level curation (L1) is the most often performed, while the more involved data level curation (L4) is more rarely carried out.

### Other curatorial measures

The same subset of staff and directors (n = 86 or 90.5%) were invited to indicate how often they believe the repository aims to ensure that data are interoperable, reproducible, peer-reviewed, or another measure of their choice. The first three concepts were defined in the survey:

- Interoperable: data are harmonized/normalized/enhanced in such a way to make them interoperable with other data in the repository. The aim is often to provide a rich resource for meta-level investigations across datasets.
- Reproducible: data and code are examined in order to reproduce the results presented in an associated scholarly work or article.
- Peer-reviewed: peer researcher from the same domain as the author reviews the data.

**Fig 3:**
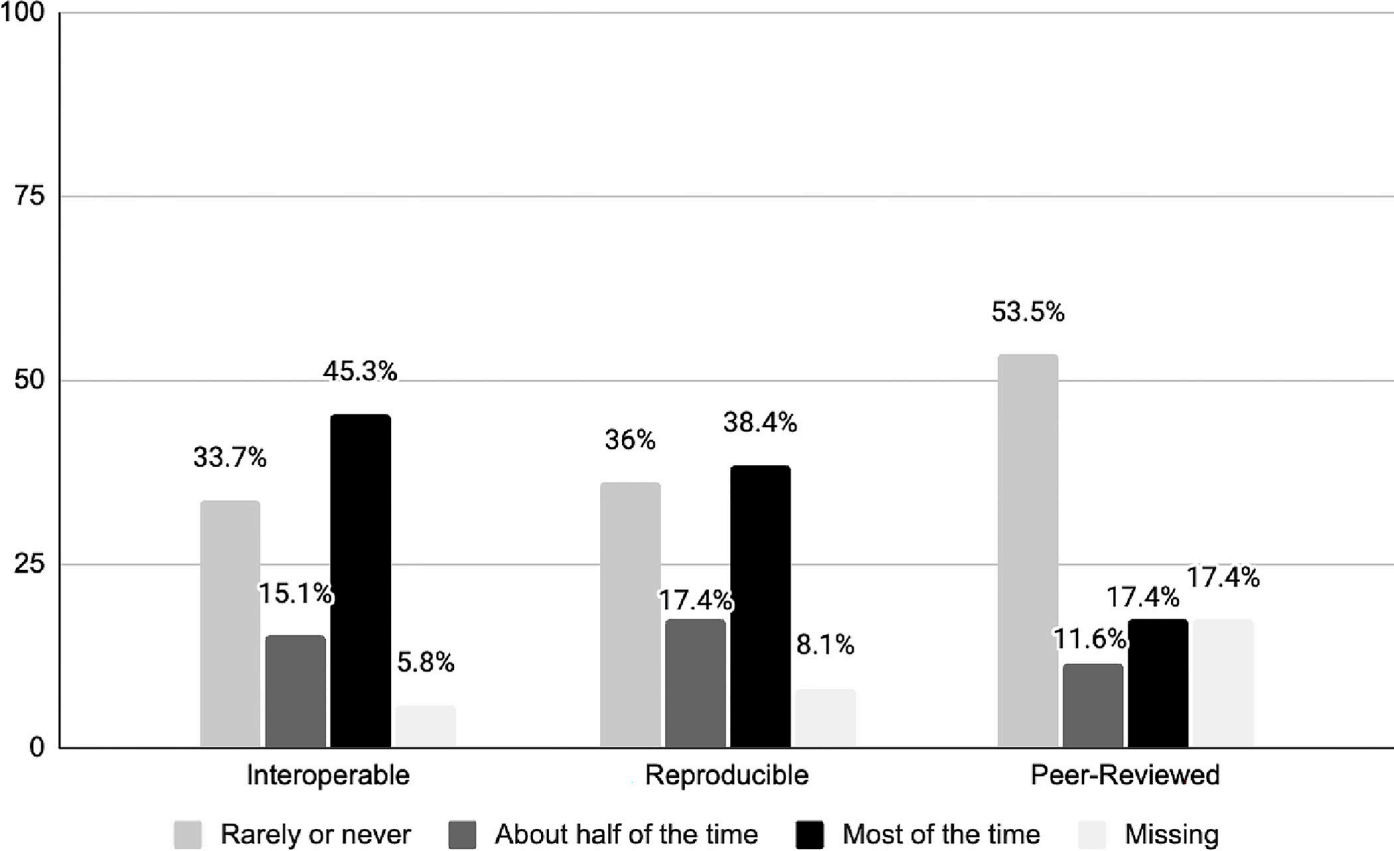
Frequency with which actions related to interoperability, reproducibility, or peer-review are performed.

The majority of the respondents indicated their repository took steps to make data interoperable and reproducible at least half the time (Fig 3). In contrast, only 29% of respondents believed that their repositories perform peer-review of the data at least more than half of the time. These results indicate that there is still an opportunity for repositories not only to include interoperability, reproducibility, or peer-review as a part of their curation workflow, but also to offer them more regularly as a part of their curation processes. In addition, 19.8% (n = 17) of respondents suggested “other” measures that their repository aims for. Several participant-defined measures were suggestive of the FAIR principles: reusable (five mentions), findable (three mentions), and accessible (one mention). Additional measures mentioned once were author-reviewed, transparency, responsibly shared, comprehensible, well-documented, and schema validated.

When responses associated with disciplinary and institutional repositories were compared, responses related to disciplinary repositories more often indicated an aim of interoperability (65.9%) and peer-review (36.4%) at least half of the time, than responses associated with institutional repositories (51.3% and 16.2%, respectively). Both repository types aim for reproducibility to a similar extent, with 52.3% for disciplinary, and 52.6% for institutional repositories.

### Frequency of curation actions

Those who self-identified as repository staff and directors (n = 86 or 90.5%) were asked about their general perception about the frequency with which repositories perform 27 different curation actions (when applicable), broken out by level on a 3-point Likert scale. The survey question noted that some actions may only be taken with the originating author’s approval. In absolute numbers, the top three curation actions performed “most of the time” according to participants are: *Check for duplicate files* (n = 74), *Mint persistent identifiers or PIDs* (n = 73), and *Check for corrupt/broken files* (n = 70).

We computed the results for items by aggregating their scores for “most of the time” and “about half of the time” and their percentage relative to the total valid cases for each item (Table 2). We found that *Check for duplicate files*, *Review documentation*, and *Review metadata for accuracy* are the top three actions taken according to more than about 91% of staff and directors’ perceptions. Among the data level (L4), *Open data files using appropriate software* was the most frequently performed with 86.9%, while *Reviewing data for accuracy or quality* (57.3%, 60.2%) and *Editing data for accuracy* or *quality* (34.9% each), were perceived to be less frequently taken by repositories. This divergence observed among the levels suggests the need to reframe how curation actions are grouped and how curation levels are compared.

**Table 2:** Curation actions reported to be performed at least half the time when applicable, ordered by curation level then overall rank across all actions based on percent of responses.

Level	Action	About half of the time	Most of the time	Sum	N	%	Overall rank
L1	Review metadata for accuracy	9	68	77	84	91.7	2
	Review metadata for quality	8	68	76	83	91.6	3
	Mint PIDs	2	73	75	83	90.4	4
	Edit and add metadata	7	67	74	85	87.1	7
	Add linkages	14	56	70	83	91.6	9
L2	Check for duplicate files	5	74	79	85	92.9	1
	Check for corrupt/broken files	5	70	75	85	88.2	5
	Inventory files	10	58	68	80	85.0	10
	Check for and request missing files	8	64	72	85	84.7	11
	Check for locked/encrypted files	3	62	65	82	79.3	14
	Rearrange files	19	37	56	80	70.0	15
	Transform files to alternative file formats	17	37	54	83	65.1	18
	Rename files	13	37	50	83	60.2	20
	Virus check	3	35	38	72	52.8	22
L3	Review documentation	10	68	78	84	92.9	1
	Check for/request missing documentation	9	65	74	84	88.1	6
	Verify variables/codes used in the data are defined	12	54	66	83	79.5	13
	Create documentation	13	41	54	84	64.3	19
L4	Open data files using appropriate software	12	61	73	84	86.9	8
	Review/mitigate disclosure risk or PII	5	63	68	83	81.9	12
	Review participant consent agreements	5	52	57	82	69.5	16
	Review/mitigate legal risks	7	46	53	79	67.1	17
	Review data for quality	13	37	50	83	60.2	20
	Review data for accuracy	10	37	47	82	57.3	21
	Test/run code	12	40	42	81	51.9	23
	Edit data for quality	6	23	29	83	34.9	24
	Edit data for accuracy	8	21	29	83	34.9	24

L1 = record level curation, L2 = file level curation, L3 = documentation level curation, L4 = data level curation

We computed the mean scores followed by a Mann-Whitney U test to verify whether institutional and disciplinary repositories differ in curation actions (S2 Appendix). Results show that a total of 12 curation actions, were found to be statistically different (p≤0.05) between the two groups being four at L2—File Level (i.e., rearrange files, rename files, transform files to alternative formats and virus check), two at L3—Documentation Level (i.e., create documentation, verify variables), and the remaining six at L4—Data Level (i.e., open data using appropriate software, edit data for quality, edit data for accuracy, review data for accuracy, review data for quality and review/mitigate disclosure risk or personally identifiable information (PII)).

From the 27 curation actions surveyed 12 are reported as more frequently performed by those affiliated with disciplinary repositories. These actions vary across L2, L3 and L4, indicating that both repository types tend to perform record-level curation with more similar regularity. It is also worth noting that review data for quality and for accuracy (L4), which were actions perceived as less frequently performed in the overall analysis, are more highly observed in responses associated with disciplinary repositories.

We also asked repository directors and staff (n = 86) to describe any additional curation actions not already mentioned and heard from 30% (n = 28). Versioning was mentioned four times and creating derivatives or additional data products for enabling broader reuse was mentioned five times.

### Perception of the value of curation

**Fig 4:**
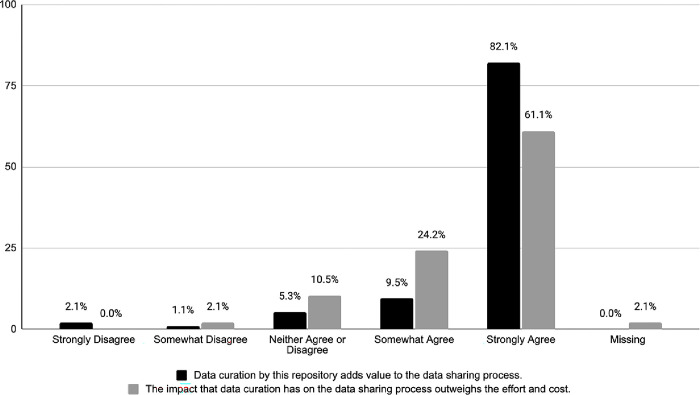
Perception of the value and impact of data curation.

The survey included several questions for all participants (n = 95) about the perceived value of curation. First, we measured participants’ agreement with the statement on a five-point Likert scale: “Data curation by this repository adds value to the data sharing process.” As shown in Fig 4, the majority of the respondents (n = 78, 82.1%) strongly agreed with this statement, and another 9.5% (n = 9) somewhat agreed with it. Similarly, they were asked to indicate, on the same Likert scale, the majority of respondents (61%, n = 58) strongly agreed with the following statement: “The impact that data curation has on the data sharing process outweighs the effort and cost” whereas 24.2% (n = 23) somewhat agreed. Curiously, out of the few responses that indicated some or strong disagreement with these two statements (n = 4), three came from repository directors.

### Most “value-add” curation action

Because of the nuanced nature of data curation practice, we included several open-ended questions throughout our survey and an impressive 90% of the participants (n = 85) provided a free-text response to the question “What is the most "value-add" curation action taken by this repository? Tell us why you think so.” *Documentation* is mentioned 36 times, nearly one third of the total curation actions identified for this question, and these responses were well distributed across respondents answering for generalist, institutional, and disciplinary repositories (see Table 3). One disciplinary repository staff member succinctly explains: “Documentation—[because] providers rarely provide any.” Another response goes into more detail on the perceived value of documentation-related curation work:

“I think most of the stuff we do adds value, but I think the documentation review is critical. We frequently receive submissions of data for publication that contain very little to no documentation, and we are able to intervene and request it from depositors before their data go live. Not only does this improve the dataset they are trying to publish at the time, but it highlights for them how important documentation is going forward. When we have had repeat depositors, they have almost always included wonderful documentation the second time around (which is pretty gratifying!).”

*Institutional data repository staff member*

**Table 3:** Top themes mentioned as the most “value-add” curation action, analyzed by repository type.

Themes mentioned as “most value-add” curation action	Generalist Repository (n = 5)	Institutional Repository (n = 40)	Disciplinary Repository (n = 40)	Total (n = 85)	Percentage of overall mentions in this theme (n = 112)
Documentation	5	17	14	**36**	32.1%
Metadata	1	11	10	**22**	19.6%
Metadata, Identifiers	1	9	0	**10**	8.9%
File Checks	3	4	2	**9**	8.0%
Additional Products & Tools	0	0	7	**7**	6.3%
PII/Confidentiality	2	0	5	**7**	6.3%
File Transformations	0	1	5	**6**	5.4%
Organization	0	3	2	**5**	4.5%
other	2	2	6	**10**	8.9%
**Grand Total**	**14**	**47**	**51**	**112**	**100.0%**

Next most commonly cited as the most value added curation action was *Metadata*, mentioned 22 times (19.6% of the themes in this qualitative analysis), but interestingly its subcategory *Identifiers*, accounting for 8.9% of the curation actions was mentioned only by respondents identifying with institutional and generalist repositories (9 and 1 mentions respectively). Disciplinary repository respondents cited creating *Additional Products or Tools* as the primary value add from their curation work. For example, one disciplinary repository staff member considered the most value-add curation action to be “Converting original data into multiple formats (e.g. Stata, SAS, SPSS, R, text)” and goes on to explain: “While we also strive for complete metadata records, distributing the data in different formats allows users to access the data in a format they feel comfortable using”.

### Perception of the impact of curation

To better understand the possible positive outcomes curation activities might produce, we asked participants to indicate their level of agreement in a five-point Likert scale in relation to 14 potential impacts. Respondents expressed high agreement for the great majority of the items indicating a general agreement of the benefit curation actions have on data value and (re)use. The only exception was to the perceived impact on data’s monetary value which received a neutral score (M = 3). Amongst the most impactful items according to respondents are the ability for others to use, find, understand, access and analyze the data, which are more closely relate to the FAIR principles.

Additionally, six respondents included other perceived impacts not listed in the survey, including: “building … collaborative relationship [sic] between repository staff and data providers” (mentioned by a disciplinary data repository director), and “protecting against identifying information/copyright” (mentioned by an unaffiliated user of a generalist data repository).

When we contrasted responses from staff and repository directors, none of the impacts listed above were found to be statistically significant, meaning that we cannot assume that the perceived impact differs between these two categories of affiliates. Nonetheless, when we compared how these impacts were perceived by those responses in reference to institutional and disciplinary repositories, by employing Mann-Whitney U tests, we could observe statistically significant difference between groups on eight out of the 14 items, namely: number of times the data is downloaded (u = 432, p < .001), uniqueness of the data (u = 568, p = .001), monetary value of the data (u = 569, p = .002), ability for the data to be aggregated with other data (u = 484.5, p < .001), ability for others to analyze the data (u = 512.5, p < .001), ability for others to understand the data (u = 718.5, p = .0026), ability for others to use the data (u = 649.5, p = .001), and portability of the data into new formats or software (u = 610, p = .003) (S3 Appendix).

### Divergence among responses associated with the same repository

As previously mentioned, 11 repositories represented in our sample received more than one response. We decided to treat them as individual responses given that most questions were framed around perceptions, and that an attempt to reconcile responses could be arbitrary.

By inspecting variation in responses for three of the repositories that received the highest number of multiple responses, we observed a high variability (coefficient of variance (CV)>1) for 18 of the 73 items measured by the survey. Additionally, responses from these three repositories were characterized by variation in the perceived frequency at which L0 (distributed as deposited), L1 (Record-level), and L2 (Documentation-level) curation was performed. In addition, three areas consistently diverged across the three repositories (with variance between 2.1 and 21.3) with respect to frequency with which curation is performed at L1 Record-level and L2 Documentation-level, and whether the data are distributed as deposited (L0).

While further research is needed to better explore these existing discrepancies in views from different staff from the same repository, we assume that individuals’ perceptions and awareness about the offering of curatorial activities, and specifically the frequency with each of these are performed, are influenced by individual past experiences or active engagement in such tasks. We recognize that getting an authoritative answer about the breadth of possible curation actions and how regularly they are performed is not an easy task, which prompts the discussion of how further studies could better handle data collection considering the repository-level as the unit of analysis.

### Qualitative themes

A total of 60 codes emerged from our qualitative analysis—grouped into six main topics of Curation Actions, Engagement, Goals, Impact, Limitations, and Workflow (Table 4).

**Table 4:** Themes, codes, definitions, and counts of responses for each resulting from free text response qualitative analysis.

Theme	Code	Description	Count of responses with this code
**Curation Actions**	documentation	reviewing, checking or verifying documentation, including readme files	48
	metadata	reviewing, checking or verifying metadata content and/or links	32
	file checks	examining data or metadata files (e.g. file format validation, virus checks, addressing missing values or codes)	27
	PII/confidentiality	handling of human subject or other sensitive types of data	18
	metadata-identifiers	adding a persistent identifier, such as a DOI	14
	additional products and tools	creating derivatives of the data submitted to them, and/or offering tools for analysis within repository system	13
	file transformations	transforming file format (e.g. creation of a stats package)	12
	metadata-standards-community	using a community standard	10
	IP/copyright/licensing	examining issues around IP, copyright and/or licensing	7
	organization	data or file organization (e.g. structure, file naming etc.)	6
	code runs	ensuring that code runs	5
	validation	validating metadata or data	5
	metadata-standards-local	using a local, possibly repository-specific metadata standard	4
	versioning	versioning of files	4
	reproduce analysis	process of fully reproducing analysis	3
**Engagement**	depositor	with person(s) who deposited the data into the repository	40
	community	with the community (e.g. best practices, training, etc.)	5
	librarians	with library staff (e.g. liaisons)	4
	peer review	with a peer-reviewer	2
	publishers	with publishers	1
**Goals**	use	to facilitate use and/or reuse	37
	discovery	to ensure the data is discoverable	25
	access	to provide access to the data	14
	quality	data/metadata would meet a specified level of quality, is subject to quality control, or that errors found would be noted or addressed	14
	interoperability	interoperability, including machine readability	11
	understandability	that a user work be able to understand or comprehend the data set	10
	completeness	to ensure data and/or metadata is "complete" (e.g. includes any necessary software used for processing)	8
	preservation	to ensure longevity of the data and/or permanent storage	7
	education	to educate or teach depositors good practices around data handling and sharing	6
	FAIR	to conform to the FAIR standard	6
	reproducibility	to ensure reproducibility	6
	compliance-format	data or metadata compliance with a specific set of requirements for formatting or structural standards	4
	low barriers	to make things as easy and inexpensive as possible for the researchers, both depositors and users	4
	use-cross-disciplinary	cross disciplinary use, or use outside of the original discipline	4
	data citation	to promote the value of data as a research output	3
	discovery-links	to enable discovery by the provision of links to/from the record	3
	compliance	to ensure compliance with. . .	2
	integrity	to ensure researcher integrity	2
	transparency	transparency	2
	responsible sharing	to ensure data is shared responsibly	1
**Impact**	use	impact on use and/or reuse, including other disciplines	6
	security	impact on security or safety	4
	data analysis	impact on data processing or analysis	3
	IP/Copyright	impact on protection of IP / copyright	2
**Limitations**	resources	not enough capacity in some area, or needing more recourse, financial or human	6
	expertise	lack of skills or expertise	4
	lack of information	lack of information hinders curation work or utility of the data	3
	technical capacity	technical capacity (e.g. size of dataset, number of files)	3
	authority	lacking authority to make changes to the data or ask for changes to be made	2
	peer review	desire but inability to do peer review	2
	policy	policy, or lack of policy, hampers curation work or ability to achieve goal	2
**Workflow**	multiple levels of curation	they have different levels of curation requiring different workflows/tasks	11
	data/file level of curation	curation at the data or file level, including file transformations	9
	automation	automation or machine-based curation activities	6
	workflow	workflow or plan that curators follow	6
	timing-intake	curation action was taken at dataset intake	3
	timing-regularity	curation happening on a regular schedule or at multiple times	3
	work level of curation	curation done at work (or item) level	3

Responses were counted for a code if the topic was mentioned, including if the comment refers to *not* doing it. A single response could be associated with more than one code.

#### Theme: Curation actions

A total of 15 codes related to *Curation Actions* emerged from the free text responses; 208 statements were associated with those codes. Overall, the most common codes in this theme were related to reviewing, checking, or verifying *Documentation*, such as readme files (mentioned 48 times) and *Metadata* (32 mentions). For example,

“All metadata are reviewed. Metadata links to data are validated and verified. File formats, experimental techniques etc.. are validated against the data uploaded. Metadata is required to be in a standard format such as ISA-TAB.”

*Disciplinary data repository staff member*

On the other hand, a few respondents used the free-text fields to mention curation actions they were *not* taking, including 1–2 mentions of *Additional Products and Tools*, *Code Runs*, *Reproduce Analysis*, *IP/Copyright*, *PII/Confidentiality*, or, in one instance, not doing curation at all.

#### Theme: Curation workflow

The idea of a *Workflow* or plan that curators follow in their work was also identified as a theme in the free text responses; seven codes were identified under this theme. Overall, disciplinary repositories mentioned *Workflows* most often (26 of 42 mentions), with mentions of *Multiple Levels of Curation* and *Data/File Curation Work* being most common. The theme of *Automation* of curation workflows was mentioned by six different responses, for example:

“Data collection is… checked and verified before being submitted to repository [sic] then run through extensive computerized error checks (range, consistency, within form/visit and across form/visit and others).”

*Disciplinary data repository director*

#### Theme: Engagement

The idea that the actions of the curators either directly engage, or enable *Engagement* with, a group or groups was also identified as a common theme, with 53 mentions in the responses. Engagement with the *Depositor* (40 mentions), seemed to be important both to ensure the quality of the work (“we think data curation must be connected with scientist [sic] work together to find deeper problems”), but also, benefits to the relationship between the repository or repository staff and submitter are mentioned, as explained here:

“Direct communicatio [sic] with the author creates a solid relationship and therefore allows the library to work with them on other data management needs. We can improve their data workflows using a real example and their data is better organized and described the next time the deposit to the repository.”

*Institutional data repository director*

The idea of *Engagement* with either depositors, library staff, the community or with users was most commonly mentioned in the responses by institutional repositories (28 of 53 mentions).

#### Themes: Goals / impact

The free text responses to the survey included multiple statements addressing the goals or the intended impacts of the data curation work. The most frequent statements made by survey respondents on *Goals* and *Impacts* addressed 3 of the 4 elements of the FAIR principles, with *Use* receiving 45 total mentions (across both themes), *Discovery* (or *Linkages* related to *Discovery*) 28, and *Access* 14 mentions. *Interoperability* was mentioned in both the positive sense (9 mentions) and twice that it was not something that was done. The *FAIR* principles themselves were mentioned as a goal six times, with one respondent stating that their repository did not yet meet their goals for FAIR data.

In examining the content of these statements on goals, there was a particular emphasis on obtaining robust documentation, developing the metadata and assigning identifiers in curation work as a means to support discovery, access and use of the data. One respondent sums it up:

“From my perspective: Documentation, metadata, and reusability evaluation to help researchers ensure that their data can actually be found and re-used by people who download it. To me, this helps foster a larger effort to promote data sharing as something genuinely useful and not just an administrative/publishing burden. For researchers, it’s pretty clear that they view the DOI minting as the biggest value-add.”

- *Institutional data repository director*

Respondents also indicated the importance of enhancing the quality of the data they curate as a goal (14 mentions). Respondents also mentioned several goals relating to quality such as *Underst*andability (10 mentions), *Completeness* (8 mentions), and *Integrity* of the data (2 mentions). Responses that referred to *Quality* tended to talk more about the data itself rather than metadata or documentation and came from respondents who were employed by disciplinary repositories much more so than those at institutional or generalist repositories. In contrast, curators who worked at institutional repositories were more likely to address the *Understandability* of the data as a goal than those who were associated with disciplinary repositories.

#### Theme: Limitations

In addition to discussing the value of their work, some of the curators’ responses revealed what they see as *Limitations* in their abilities or in the services they offer. A lack of *Resources* was the most commonly mentioned limitation (6 mentions) and was expressed equally by disciplinary, generalist and institutional repositories. *Staffing Levels* and the *Scalability* of curation practices were among the concerns expressed by curators, as expressed in this response:

“We are a small repository started 3 years ago. As one person, I have limited capacity to review and curate with my other duties in Research Data management. At the same time, my goal is to grow awareness of the repository and make the deposit process low barrier to entry for researchers. With this in mind, while I would like to provide a higher level of curation services, I do this work by researcher request, and try to educate researchers at the outset of their work. I hope that there will be more capacity and willingness in the future to do more curation, but at this time, just getting folks to use the repository is the biggest hurdle we face.”

- *Institutional data repository director*

## Discussion

### Levels of curation are multi-faceted

While “levels of curation” as a model to help understand current practices within a repository is useful and may help orient end-users and potential depositors to these complex services, our results indicate that describing a repository as providing “a level” of curation is problematic. For example, while a majority of repositories (74%) in our sample were said to perform curation at the data-level (L4) in our survey, the least likely curation action to be performed was *L4—Editing data for quality* and L4—*Editing data for accuracy* (34% each); however, the data-level action of opening data files with the appropriate software was reported by 86.9% of respondents. Data-level curation can involve a variety of actions. For instance, while a repository might "open data files with the appropriate software" and do a general review for quality (such as checking for missing data), which are considered a "data-level" actions, they may not go deeper to actually edit the data. Two respondents explain:

“Within certain activity categories, some actions are always performed (e.g., data files are always opened and previewed, saved in various formats) but others are rarely or never performed (e.g., edits to the data is almost never done).”

Disciplinary data repository staff member

“While our repository tries to curate at the data/file level whenever possible, there are times when… the nature of the data is such that it’s not feasible to curate at a deeper level (depending on the size of the dataset, number/format of the files).”

*- Institutional data repository staff member*

*We anticipated that there would be variation in curation actions and how those coincide with a stated level for a few reasons*. Differences in datasets, such as the format or subject area, could be anticipated to impact curatorial efforts locally. Curation at “higher” levels requires resources and expertise that are not necessarily available at every repository participating in the survey, and the scope of the collection for each repository will affect both staffing and resources. Likewise, the willingness of researchers to work with a curator to enhance a given dataset affects the results of the curatorial process. As funding agencies increasingly view curation as a desirable characteristic for repositories [39, 40], having methods to conceptualize and operationalize curation services within a given repository will help inform decisions by depositors and facilitate transparency with stakeholders.

### Some curation actions are more common than others

**Fig 5:**
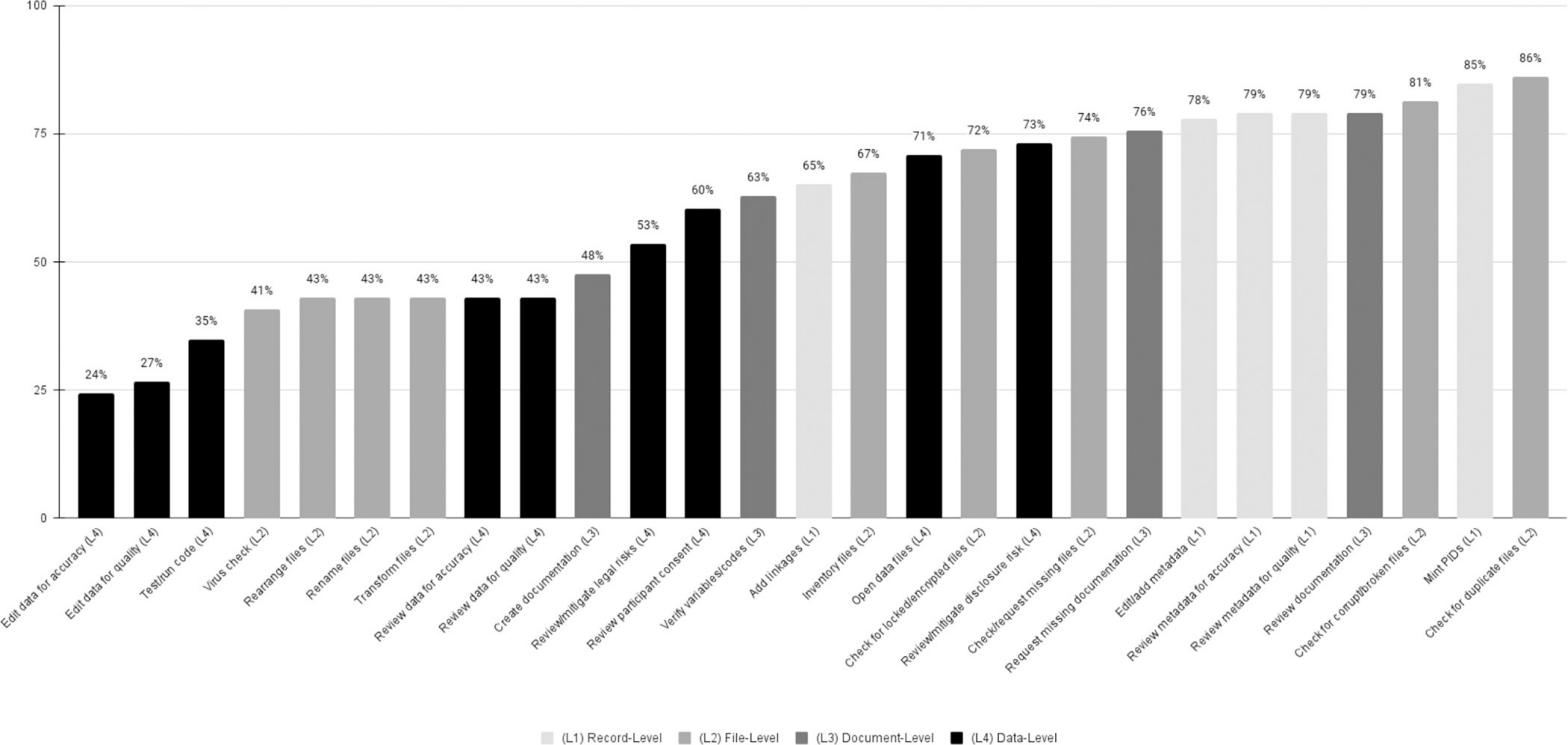
Frequency of curation actions reported as performed most of the time (n = 86).

One purpose of our research was to more fully understand the scope of specific curation actions that may be performed and that could be benchmarked for future research, as well as to inform repositories’ practices. Therefore, we decided to reimagine our proposed category of curatorial levels, which initially considered the type and focus of work (documentation, record, file, or data), by visualizing the data according to their frequency performed (most of the time) as shown in Fig 5.

This view of the data allows us to visualize actions that most repositories in our sample were providing while also seeing the breadth in scope as associated with the four “levels” of curation. There appears to be a trend, unsurprisingly, that actions that are highly specialized and work-intensive actions, such as *edit data for quality* and *accuracy*, are less frequently performed. As we consider the resource implications for making decisions regarding what services to provide, we can use these types of benchmarks to place our own work in the context of others and to acknowledge the resource implications of these decisions. While it is outside the scope of this paper to make specific recommendations for which curation actions should be provided or prioritized, from our findings it appears most repositories see particular value in assisting researchers with the creation and enhancements of documentation and metadata. Likewise, identifying the actions less likely performed can help inform future research into what processes may benefit from additional automation or training.

### Perception of the levels of curation differs across repository type

The differences of the frequency of performing certain curation actions between disciplinary and institutional repositories are also notable. We found that staff in disciplinary repositories said they perform 12 out of 27 actions more frequently than our sample of institutional repositories, with *renaming files*, *creating documentation*, editing *data for quality and accuracy* and reviewing data for accuracy and quality having the highest significant divergence (p ≤ .001). Likewise, disciplinary repositories indicated a higher frequency of performing interoperability and peer-review actions than institutional repositories. While these findings may be interpreted to reinforce a general perception that disciplinary repositories have more depth of subject-specific expertise, thereby affecting their ability to perform certain curation actions more often, for the majority of surveyed the curation actions we could not assume statistically significant divergences between disciplinary and institutional repositories.

### Perception of the levels of curation differs across staff at the same repository

Like levels of curation, we observed differences among responses for the same repository on the frequency of curation actions taken. These differences could be due to how staff and directors of repositories may or may not be aware of the full range of curation actions taken. For example, a repository director may be aware of curation actions that are automated by the system (e.g., virus checking). However, different data types may require different actions that only curators close to the work may be aware of. Repositories may also provide certain services or activities, such as peer review, based on requests or needs of particular stakeholders (such as journals that require data to be examined during peer review) and different curators may handle these requests independently. Onboarding, cross-training curation staff may benefit from including a thorough overview of the full curation approaches taken at a repository and the further development of standard operating procedures (SOPs) within a repository. Finally, it is critical that repositories clearly document and communicate these SOPs to end-users for transparency in process as well as to receive acknowledgement for curatorial efforts. More research is needed to reach an authoritative answer about the breadth of actions across repository types and how regularly they are performed, considering the repository-level as the unit of analysis.

### Despite the complexity, most perceive the value of curation

Another goal of our research was to determine the value-add that curation has on the data sharing process. Exploring not only what processes repositories perform but *why* they commit resources to curation. Perhaps unsurprisingly, most of the respondents strongly agree that the actions taken by curators at repositories add value to the datasets published in repositories. We found both in the quantitative and qualitative analysis that the highest perceived impact of curation resonates with the FAIR principles and the ability for others to use (95%), find (95%), access (93%), and understand (92%) data, reinforcing the idea that our ultimate goal of curation is improving the reuse and quality of datasets. The importance of reviewing documentation was a trend found both as one of the most common curation actions (90%) and was also most often mentioned in the qualitative responses related to the most “value-add” curation actions.

However, our qualitative analysis highlighted that the value of curation goes beyond the mechanics of making data more FAIR but also has broader social and educational benefits such as spurring engagement with researchers and educating researchers in good data sharing practices. While the curator community sees the value of curation, they also raise concerns related to the realities of scalability and expertise. Certain curation aims (including reproducibility and interoperability) may remain outside the scope for many repositories due to resource limitations. Although we note that over 50% of our respondents reported curating for reproducibility at least half the time, this question was phrased broadly and more follow-up research would be needed to understand exactly how those repositories perform those actions. Likewise, more research is needed to understand researcher’s perception of the value of curation and how those might differ or compliment those of repository staff. A companion study performed by the Data Curation Network with depositors to six institutional repositories provides a more comprehensive view of the value of curation across stakeholders [37].

### Study limitations and future research

The self-selection approach of survey distribution presents a known risk of bias and possibly data skewness [41], towards staff that work at repositories that perform curation. Due to respondents being part of the repository community, there is potential bias in our sample toward seeing value in curation work overall, however this perception of value is echoed by researchers in a companion study [37]. We also acknowledge that our limited sample and national focus restricts the generalizability of our results. We see opportunities for future study which expands the scope to international data curation practices and takes a more targeted sampling approach. Because this survey was exploratory in nature, we also see possibilities for new studies to verify the reliability of scales and survey items for the purpose of conducting more inferential and parametric statistics to test relationships of constructs based on an underlying nomological network.

## Conclusions

This survey focused primarily on the reported curation practices and activities of repositories providing access to data and perceptions related to the value of these curation activities. Understanding the current actions or “levels” of curation being provided within US-based repositories provides useful benchmarking data as repositories assess their current services with an eye towards what are the most common practices and what are the most impactful. Variation of curation actions can be influenced by the resources of the repository, the expertise needed to perform a particular activity, and the research communities the repositories serve. Transparency of curation practices will aid repositories in developing and communicating standard procedures as well as raise awareness among key stakeholders—including users, funders, and the research community—of the important role that curators play. While it may not be surprising that those working within repositories believe that data curation adds value to the data sharing process or that it potentially positively impacts the ability for others to use, find, understand, and access the data, repository staff and directors are in a unique position to assess this potential value proposition as they view the data before and after the curation process and can provide rich experiential data for informing our understanding of the value of curation. As data repositories continue to grow in importance with advancements in and requirements around public access, research into the breadth and scope of curation practices provides practical applications for policy-makers and repository managers.

## Supporting information

S1 Appendix 59 US data repositories represented in survey results. (PDF)

S2 Appendix Descriptive statistics and Mann-Whitney U test of curation actions performed. (PDF)

S3 Appendix Descriptive statistics and Mann-Whitney U tests of potential impacts. (PDF)
