# Supplementary material for: Understanding the value of curation: A survey of US data repository curation practices and perceptions
Source: PLoS One. 2024 Jun 14;19(6):e0301171. doi: 10.1371/journal.pone.0301171 (PMC11178225; doi:10.1371/journal.pone.0301171)
Supplement: S1 Appendix — (PDF) [file pone.0301171.s001.pdf]

S1 Appendix: 59 US data repositories represented in survey results.

Repository Name	URL
AphasiaBank	https://aphasia.talkbank.org/
Arizona State University Library Research Data Repository (ASU Dataverse)	http://dataverse.asu.edu
Biological and Chemical Oceanography Data Management Office (BCO-DMO)	http://www.bco-dmo.org
California Institute of Technology Research Data Repository (CaltechDATA)	http://data.caltech.edu
California State University (CSU) ScholarWorks	http://scholarworks.calstate.edu/
Carnegie Mellon University (CMU) kilthub	http://kilthub.cmu.edu/
Center for Strategic Scientific Initiatives (CSSI) Data Coordinating Center (CSSI DCC)	http://cssi-dcc.nci.nih.gov
ClinVar	https://www.ncbi.nlm.nih.gov/clinvar/
Cornell University Library eCommons	https://ecommons.cornell.edu/
Data and Specimen Hub (DASH)	https://dash.nichd.nih.gov/
Data and Statistical Services, Princeton University Library	https://dss.princeton.edu/
Data Repository for University of Minnesota (DRUM)	http://conservancy.umn.edu/DRUM
database of Genotypes and Phenotypes (dbGaP)	https://www.ncbi.nlm.nih.gov/gap/
DataSpace at Princeton University	http://dataspace.princeton.edu
Digital Commons at USU	https://digitalcommons.usu.edu/
Division of Intramural Population Health Research (DIPHR)	https://brads.nichd.nih.gov/
Dryad	https://datadryad.org/
DSpace@MIT	http://dspace.mit.edu
Duke University Research Data Repository	https://research.repository.duke.edu/
Earth Observing System Data and Information System (Earthdata)	http://earthdata.nasa.gov
EarthChem Library	https://www.earthchem.org
Emory Dataverse	https://dataverse.unc.edu/dataverse/emory
Federal Interagency Traumatic Brain Injury Research (FITBIR)	http://FITBIR.nih.gov
Florida Fish and Wildlife Research Institute (FWC Digital Library)	http://f50006a.eos-intl.net/F50006A/OPAC/
gene Expression Analysis Resource (gEAR)	http://umgear.org/
Harvard Dataverse	http://dataverse.harvard.edu
Immport Shared Data	http://immport.org

Inter-university Consortium for Political and Social Research (ICPSR)	http://www.icpsr.umich.edu
Johns Hopkins University (JHU) Data Archive	https://archive.data.jhu.edu
UVA LibraData	http://dataverse.lib.virginia.edu
Microsoft Data Platform	https://www.microsoft.com/zh-cn/sql-server/
Mountain Scholar, Data - Colorado State University	https://mountainscholar.org/handle/10217/172830
Mouse Phenome Database (MPD)	https://phenome.jax.org/
National Alzheimer’s Coordinating Center (NACC)	https://naccdata.org/
National Bureau of Economic Research (NBER)	http://www.nber.org/research/data
National Eye Institute (NEI) Data Commons	https://neidatacommons.nei.nih.gov/
National Institute of Neurological Disorders and Stroke (NINDS) Parkinson's Disease Biomarkers Program (PDBP)	http://pdbp.ninds.nih.gov/
NCBI Gene Expression Omnibus (GEO) Datasets	http://www.ncbi.nlm.nih.gov/gds
New York University (NYU) Faculty Digital Archive	http://archive.nyu.edu
NIH Figshare	http://nih.figshare.com/
Oak Ridge National Laboratory Distributed Active Archive Center (ORNL DAAC) for Biogeochemical Dynamics	http://daac.ornl.gov
Penn State University Scholarsphere	http://scholarsphere.psu.edu
Qualitative Data Repository (QDR)	https://qdr.syr.edu
Scholarship@Miami	http://scholarship.miami.edu
SEAD	https://sead2.ncsa.illinois.edu
Socioeconomic Data and Applications Center (SEDAC)	https://sedac.ciesin.columbia.edu/
TalkBank	http://talkbank.org
University of Arizona Research Data Repository (Arizona ReData)	http://arizona.figshare.com
University of California San Diego (UCSD) Library Digital Collections	https://library.ucsd.edu/dc
University of Houston (UH) Dataverse	https://dataverse.tdl.org/dataverse/uh
University of Illinois Data Bank (Illinois Data Bank)	http://databank.illinois.edu
University of Maryland Baltimore (UMB) Data Catalog	https://datacatalog.hshsl.umaryland.edu/
University of Michigan Deep Blue Data (Deep Blue)	https://deepblue.lib.umich.edu/data/
University of North Carolina (UNC) Dataverse	https://dataverse.unc.edu
University of Oregon Scholars' Bank (Dataverse @ University of Oregon)	https://dataverse.harvard.edu/dataverse/scholarsbankdata
University of Oregon Scholars' Bank	https://scholarsbank.uoregon.edu/xmlui/

University of Pittsburgh D-Scholarship@Pitt	http://d-scholarship.pitt.edu/
University of Wyoming (UW) Research Data	https://mountainscholar.org/handle/20.500.11919/3204
Washington University in St. Louis (WUSL) Open Scholarship	https://openscholarship.wustl.edu/
