# Supplementary material for: Understanding the value of curation: A survey of US data repository curation practices and perceptions
Source: PLoS One. 2024 Jun 14;19(6):e0301171. doi: 10.1371/journal.pone.0301171 (PMC11178225; doi:10.1371/journal.pone.0301171)
Supplement: S2 Appendix — (PDF) [file pone.0301171.s002.pdf]

S2 Appendix - Curation Action Results

Table A: Descriptive Statistics for Curation Actions (All Cases Included)

Levels	*Curation Action*	*N*	*m*	*M*	*SD*	Var(X)	Min	Max
(L1)	Add linkages	82	2.5	3.0	0.7	0.5	1.0	3.0
	Edit/add metadata	85	2.7	3.0	0.7	0.5	1.0	3.0
	Mint persistent identifiers	83	2.8	3.0	0.6	0.4	1.0	3.0
	Review metadata for accuracy	84	2.7	3.0	0.6	0.4	1.0	3.0
	Review metadata for quality	83	2.7	3.0	0.6	0.4	1.0	3.0
(L2)	Check for and request missing files	85	2.6	3.0	0.7	0.6	1.0	3.0
	Check for corrupt/broken files	85	2.7	3.0	0.7	0.4	1.0	3.0
	Check for duplicate files	85	2.8	3.0	0.6	0.3	1.0	3.0
	Check for locked/encrypted files	82	2.6	3.0	0.8	0.7	1.0	3.0
	Inventory files	80	2.6	3.0	0.7	0.6	1.0	3.0
	Rearrange files	80	2.2	2.0	0.9	0.7	1.0	3.0
	Rename files	83	2.1	2.0	0.9	0.9	1.0	3.0
	Transform files to alternative file formats	83	2.1	2.0	0.9	0.8	1.0	3.0
	Virus check	72	2.0	2.0	1.0	1.0	1.0	3.0
(L3)	Check for and request missing documentation	84	2.7	3.0	0.7	0.5	1.0	3.0
	Create documentation	84	2.1	2.0	0.9	0.8	1.0	3.0
	Review documentation	84	2.7	3.0	0.6	0.3	1.0	3.0
	Verify variables/codes	83	2.5	3.0	0.8	0.7	1.0	3.0
(L4)	Open data files using appropriate software	84	2.6	3.0	0.7	0.5	1.0	3.0
	Edit data for quality	83	1.6	1.0	0.9	0.8	1.0	3.0
	Edit data for accuracy	83	1.6	1.0	0.9	0.8	1.0	3.0
	Review data for accuracy	82	2.0	2.0	0.9	0.9	1.0	3.0
	Review data for quality	83	2.1	2.0	0.9	0.9	1.0	3.0
	Review/mitigate disclosure risk or PII	83	2.6	3.0	0.8	0.6	1.0	3.0
	Review/mitigate legal risks e.g., copyright check	79	2.3	3.0	0.9	0.9	1.0	3.0
	Review participant consent agreements	82	2.3	3.0	0.9	0.8	1.0	3.0
	Test/run code	81	1.9	2.0	0.9	0.9	1.0	3.0

Table B: Mean and Median Scores for Curation Actions by Repository Type

		Disciplinary		Institutional	
**Levels**	***Curation Action***	***m***	***M***	***m***	***M***
(L1)	Add linkages	2.53	3.0	2.63	3.0
	Edit/add metadata	2.74	3.0	2.56	3.0
	Mint persistent identifiers	2.70	3.0	2.88	3.0
	Review metadata for accuracy	2.78	3.0	2.71	3.0
	Review metadata for quality	2.76	3.0	2.67	3.0
(L2)	Check for and request missing files	2.76	3.0	2.46	3.0
	Check for corrupt/broken files	2.84	3.0	2.56	3.0
	Check for duplicate files	2.87	3.0	2.71	3.0
	Check for locked/encrypted files	2.73	3.0	2.36	3.0
	Inventory files	2.65	3.0	2.50	3.0
	Rearrange files	2.54	3.0	1.97	2
	Rename files	2.49	3.0	1.78	1.5
	Transform files to alternative file formats	2.47	3.0	1.88	2.0
	Virus check	2.44	3.0	1.74	1.0
(L3)	Check for and request missing documentation	2.70	3.0	2.59	3.0
	Create documentation	2.65	3.0	1.78	2.0
	Review documentation	2.84	3.0	2.66	3.0
	Verify variables/codes	2.65	3.0	2.25	3.0
(L4)	Open data files using appropriate software	2.84	3.0	2.39	3.0
	Edit data for quality	2.14	3.0	1.20	1.0
	Edit data for accuracy	2.14	2.0	1.15	1.0
	Review data for accuracy	2.41	3.0	1.67	1.0
	Review data for quality	2.39	3.0	1.72	1
	Review/mitigate disclosure risk or PII	2.79	3.0	2.36	3
	Review/mitigate legal risks e.g., copyright check	2.26	3.0	2.18	3
	Review participant consent agreements	2.37	3.0	2.26	3
	Test/run code	1.91	2.0	1.90	2

Table C: Mann-Whitney Test (Disciplinary vs. Institutional)

Levels	*Curation Action*	Mann-Whitney U	Wilcoxon W	*Z*	*p*
(L1)	Add linkages	643.0	1309.0	-1.0	0.3
	Edit/add metadata	694.0	1555	-1.2	0.244
	Mint persistent identifiers	674.0	1377	-1.2	0.227
	Review metadata for accuracy	700.5	1561.5	-0.9	0.4
	Review metadata for quality	688.5	1468.5	-0.775	0.4
(L2)	Check for and request missing files	647.5	1508.5	-1.728	0.084
	Check for corrupt/broken files	665.0	1526	-1.685	0.092
	Check for duplicate files	690.5	1551.5	-1.444	0.149
	Check for locked/encrypted files	588.0	1368	-1.84	0.066
	Inventory files	635.0	1376	-0.93	0.352
	Rearrange files	430.0	1210	-2.976	0.003*
	Rename files	429.5	1249.5	-3.46	<.001***
	Transform files to alternative file formats	470.0	1331	-2.972	0.003**
	Virus check	345.0	940	-2.894	0.004**
(L3)	Check for and request missing documentation	699.5	1560.5	-0.802	0.422
	Create documentation	362.50	1223.5	-4.356	<.001***
	Review documentation	686.5	1547.5	-1.079	0.281
	Verify variables/codes	569.0	1389	-2.063	0.039*
(L4)	Open data files using appropriate software	527.0	1388	-2.978	0.003**
	Edit data for quality	359.5	1179.5	-4.576	<.001***
	Edit data for accuracy	322.0	1142	-5.009	<.001***
	Review data for accuracy	419.5	1199.5	-3.437	<.001***
	Review data for quality	446.5	1226.5	-3.254	0.001**
	Review/mitigate disclosure risk or PII	566	1346	-2.369	0.018**
	Review/mitigate legal risks	641	1382	-0.3	0.764
	Review participant consent agreements	682	1423	-0.491	0.623
	Test/run code	695.5	1515.5	-0.052	0.958

Asymp. Sig. (2-tailed), (*) $p < 0.05$, (**) $p < 0.01$, (***) $p < 0.001$.
