# Supplementary material for: Understanding the value of curation: A survey of US data repository curation practices and perceptions
Source: PLoS One. 2024 Jun 14;19(6):e0301171. doi: 10.1371/journal.pone.0301171 (PMC11178225; doi:10.1371/journal.pone.0301171)
Supplement: S3 Appendix — (PDF) [file pone.0301171.s003.pdf]

S3 Appendix - Potential Impact Analysis

Table A: Descriptive Statistics for Potential Impact (All Cases Included)

**Potential Impact**	***N***	***m***	***M***	***SD***	**Var(X)**	**Min**	**Max**
Number of times the data is downloaded	92	4.21	4	0.859	0.737	2	5
Uniqueness of the data	93	3.72	4	1.192	1.421	1	5
Monetary value of the data	92	3.22	3	1.21	1.469	1	5
Ability for the data to be aggregated with other data	93	4.13	4	1.01	1.027	1	5
Ability for others to find the data	93	4.7	5	0.73	0.539	1	5
Ability for others to access the data	93	4.66	5	0.63	0.402	2	5
Linkages to articles and other data	93	4.49	5	0.83	0.687	1	5
Ability for others to analyze the data	93	4.57	5	0.68	0.465	2	5
Ability for others to understand the data	92	4.66	5	0.73	0.534	1	5
Ability for others to use the data	93	4.73	5	0.51	0.264	3	5
Ability for others to trust the data	93	4.2	4	0.92	0.838	1	5
Portability of the data into new formats or software	93	4.1	4	1.14	1.306	1	5
Preservation of the data	93	4.46	5	0.96	0.925	1	5
Relationship between the repository staff and the data providers	92	4.29	5	0.90	0.803	1	5

Table B: Mean and Median Scores for Potential Impact Items by Repository Type

	Disciplinary		***Institutional***	
**Potential Impact**	***m***	***M***	***m***	***M***
Number of times the data is downloaded	4.62	5	3.81	4
Uniqueness of the data	4.12	5	3.35	3
Monetary value of the data	3.69	4	2.93	3
Ability for the data to be aggregated with other data	4.51	5	3.72	4
Ability for others to find the data	4.74	5	4.63	5
Ability for others to access the data	4.7	5	4.63	5
Linkages to articles and other data	4.53	5	4.49	5
Ability for others to analyze the data	4.86	5	4.28	4
Ability for others to understand the data	4.83	5	4.51	5
Ability for others to use the data	4.88	5	4.56	5
Ability for others to trust the data	4.37	5	4.07	4
Portability of the data into new formats or software	4.42	5	3.88	4
Preservation of the data	4.56	5	4.3	5
Relationship between the repository staff and the data providers	4.17	4	4.42	5

Table C: Mann-Whitney Test (Disciplinary vs. Institutional)

**Potential Impact**	**Mann-Whitney U**	**Wilcoxon W**	***Z***	***p***
Number of times the data is downloaded	432	1378	-4.455	<.001***
Uniqueness of the data	568	1514	-3.206	0.001**
Monetary value of the data	569	1515	-3.128	0.002**
Ability for the data to be aggregated with other data	484.5	1430.5	-4.065	<.001***
Ability for others to find the data	857	1803	-0.84	0.401
Ability for others to access the data	878	1824	-0.512	0.608
Linkages to articles and other data	901.5	1847.5	-0.236	0.813
Ability for others to analyze the data	512.5	1458.5	-4.278	<.001***
Ability for others to understand the data	718.5	1664.5	-2.231	0.026**
Ability for others to use the data	649.5	1595.5	-3.177	0.001**
Ability for others to trust the data	735	1681	-1.766	0.077
Portability of the data into new formats or software	610	1556	-2.944	0.003**
Preservation of the data	785	1731	-1.469	0.142
Relationship between the repository staff and the data providers	777	1680	-1.22	0.222

Asymp. Sig. (2-tailed), (*) p < 0.05, (**) p < 0.01, (***) p < 0.001.
